# Self-referential forces are sufficient to explain different dendritic morphologies

**DOI:** 10.3389/fninf.2013.00001

**Published:** 2013-01-30

**Authors:** Heraldo Memelli, Benjamin Torben-Nielsen, James Kozloski

**Affiliations:** ^1^Department of Computer Science, Stony Brook UniversityStony Brook, NY, USA; ^2^The Edmond and Lily Safra Center for Brain Sciences and Department of Neurobiology, The Hebrew University of JerusalemJerusalem, Israel; ^3^IBM Research Division, Computational Biology Center, IBM T.J. Watson Research CenterYorktown Heights, NY, USA

**Keywords:** dendrite, morphology, simulation, growth cone, computational, model

## Abstract

Dendritic morphology constrains brain activity, as it determines first which neuronal circuits are possible and second which dendritic computations can be performed over a neuron's inputs. It is known that a range of chemical cues can influence the final shape of dendrites during development. Here, we investigate the extent to which self-referential influences, cues generated by the neuron itself, might influence morphology. To this end, we developed a phenomenological model and algorithm to generate virtual morphologies, which are then compared to experimentally reconstructed morphologies. In the model, branching probability follows a Galton–Watson process, while the geometry is determined by “homotypic forces” exerting influence on the direction of random growth in a constrained space. We model three such homotypic forces, namely an inertial force based on membrane stiffness, a soma-oriented tropism, and a force of self-avoidance, as directional biases in the growth algorithm. With computer simulations we explored how each bias shapes neuronal morphologies. We show that based on these principles, we can generate realistic morphologies of several distinct neuronal types. We discuss the extent to which homotypic forces might influence real dendritic morphologies, and speculate about the influence of other environmental cues on neuronal shape and circuitry.

## 1. Introduction

Dendrites are beautiful arbors sprouting from the cell bodies of neurons. The shape of dendrites is of great importance to the nervous system for two interrelated reasons. First, a neuron's dendrites receive inputs from other neurons. Since dendritic morphologies define which spatial domain can be reached, they govern the connectivity of the local circuit. Second, neuronal computation takes place along the entire dendritic path between the site of the input and the site of action potential generation in the axon (Koch and Segev, 2000; London and Häusser, 2005; Torben-Nielsen and Stiefel, 2010). Dendrites exist in a wide range of sizes and shapes and their morphologies have historically been used to classify neuronal types (Hillman, 1979; Migliore and Shepherd, 2005). Moreover, dendritic trees of one neuron type can display a large variation (Soltesz, 2005), and in some cases variations in the morphology of the dendritic tree is indicative of particular neurological disorders (Irwin et al., 2000; Kaufmann and Moser, 2000). Consequently, the morphology of dendrites has been, and remains, at the core of many studies.

To assign neuronal morphologies and their traits to distinct neuronal types, invariant descriptors are required. To this end, distributions of properties are measured directly from digitally reconstructed neurons (for instance, the distribution of segment lengths). Parameterizations of these distributions (Ascoli and Krichmar, 2000; Samsonovich and Ascoli, 2005; Koene et al., 2009) or the distributions themselves (Lindsay et al., 2007; Torben-Nielsen et al., 2008) then provide these invariant descriptors of morphology. However, despite considerable success, these approaches cannot account for all experimentally observed variations in the data. Indeed, it is often impossible to construct an exhaustive statistical model because the experimental data does not contain sufficient samples, or is simply not coherent (Torben-Nielsen et al., 2008).

An interpretation thereof is that a single dendritic morphology is the result of interactions with the environment in which it grew. These interactions are *de facto* not in the reconstructed data, which is a snapshot of one of their possible outcomes. Experimental evidence backs this interpretation as it has been shown that dendrites are shaped by chemical cues in the environment (Scott and Luo, 2001; Grueber and Sagasti, 2010; Jan and Jan, 2010), and a specific class of these environmental interactions are termed *homotypic* or *self-referential*, that is, originating from the neuron itself.

At least three self-referential forces derived from these cues are experimentally and theoretically described, namely self-avoidance (Grueber et al., 2005; Marks and Burke, 2007b; Grueber and Sagasti, 2010; Jan and Jan, 2010), soma-oriented tropism (Samsonovich and Ascoli, 2003; Marks and Burke, 2007a), and mechanical stiffness, which provides a bias to the growth cone to favor straight motion (Condron and Zinn, 1997; Koene et al., 2009). In this work, we propose a novel method, extending the work of Samsonovich and Ascoli (2003) and Marks and Burke (2007b), to model these self-referential interactions that shape neuronal morphologies. The method algorithmically generates dendrograms based on a Galton–Watson process and simultaneously adds geometry that is locally determined solely by the three aforementioned directional biases. We show that based on these simple principles, virtual morphologies with high resemblance to neurons can be generated. Hence, we demonstrate *in silico* that self-referential cues are sufficient to shape dendritic morphologies realistically given an otherwise random growth process and are thereby capable of generating multiple isometric variants of a single reconstructed instance. Because self-referential cues can account for these properties, we propose their descriptions might be useful as an integral part of overall neuronal morphological descriptions.

## 2. Methods

### 2.1. Morphogenetic algorithm

**Figure 1:**
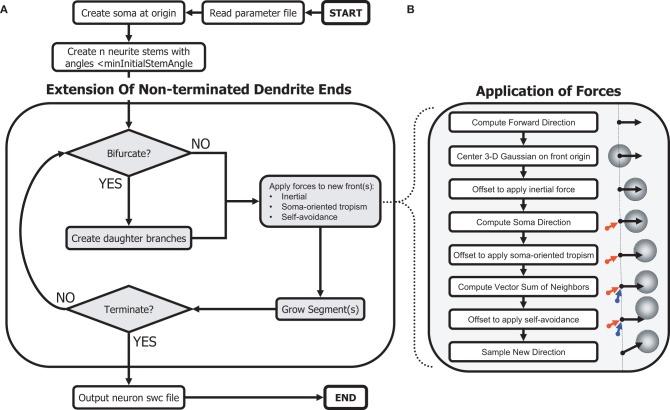
**Schematic of the morphogenetic algorithm.** The phenomenological algorithm relies on a Galton–Watson process to create a topology while the geometry results from applied forces from the environment. **(A)** Main algorithm to generate dendritic morphologies. **(B)** Procedures to sample angles biased by self-referential forces.

We present an integrated morphogenetic algorithm (Figure 1), in which a neuron's dendrogram and geometry are generated simultaneously, such that the dendrogram derives from a modified Galton–Watson process, while the local geometry is dictated solely by the sum of self-referential growth directional biases. Neurons and their branches are further subject to certain termination conditions. In addition, we include a random component of the morphogenetic process, a 3-dimensional (3-D) Gaussian distribution, from which all directions of growth are sampled.

**Figure 2:**
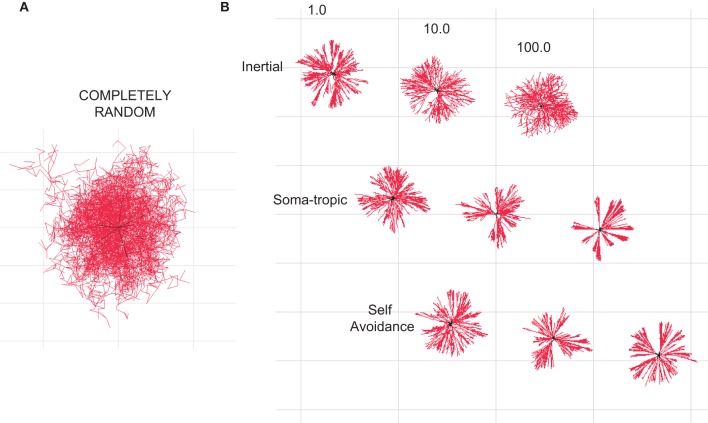
**Homotypic forces can shape dendritic morphologies.** All illustrations are 2-D projections of 3-D structures. **(A)** Branched structure resulting from a Galton–Watson branching process without homotypic forces resembles a random diffusion process. **(B)** Dendritic-zlike structures emerge when different homotypic growth biases are added to define the geometry. The influence of different levels of inertial, soma-tropic, and self-avoidance are shown. Structures are bounded by a cube with side dimensions of 50 μm.

**Figure 3:**
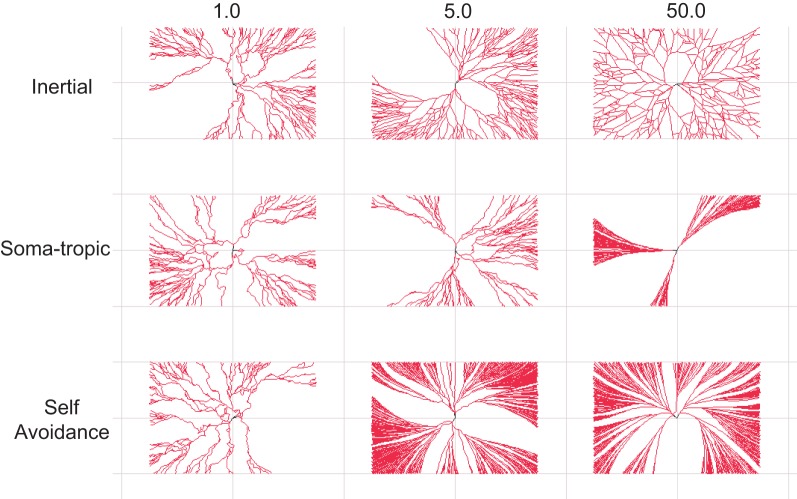
**Dendritic morphological traits associated homotypic forces.** 2-D dendritic morphologies generated with different settings for the homotypic growth biases. The modeled strength of one force is varied while the other two forces are fixed to a low strength. **Top row**: Dendrites with distinct levels of inertial forces. **Middle row**: Dendrites with distinct levels of soma-tropic forces. **Bottom row**: Dendrites with distinct levels of self-avoidance forces. Morphologies generated with strong inertial forces show sparse dendrites, while dense arbors require strong soma-tropic or self-avoidance forces. Inertial forces and self-avoidance grow larger structures in the same surface (space). Self-avoidance covers the surface (space) most densely. Structures are bounded by a rectangle with dimensions 150 × 100 μm.

A simulation begins with a specific configuration of model parameters (Table 1; we include an exemplary configuration file for each simulation in the Supplementary Materials). The main parameters of the algorithm describe the strength, spatial gradient, and extent of the local growth biases, and the branching and extension processes. A second subset of parameters describes the termination conditions for both growth of individual branches and growth of the neuron as a whole. A final subset includes auxiliary parameters for initial conditions (e.g., soma surface area, number of stems, etc.). While termination and initial conditions are typically derived directly from experimental observations, parameters governing growth biases were chosen by hand to produce morphologies that match other secondary measures such as space coverage and fractal dimension, as well as qualitative observations from experimental reconstructions. While both termination and initial conditions had a potent effect on global properties of the generated cells' morphologies (size, total fiber length, etc.), they were less able than local homotypic growth biases to alter those cellular morphological traits that often define distinct types. These traits are further demonstrated by artificial morphologies generated when holding termination and initial condition constant (Figures 2, 3).

**Table 1:** **Parameters of the morphogenetic algorithm and their explanation**.

**MAIN PARAMETERS**	
**self_avoidance_force**	Maximum strength of self-avoidance bias: 0 indicates no self-avoidance, positive/negative (unbounded) values introduce a bias
**self_avoidance_decay**	Self-avoidance force decays as a function of distance to this power
**soma_tropic_force**	Maximum strength of soma-tropic bias: 0 indicates no soma-tropic bias, positive/negative (unbounded) values introduce a bias
**soma_tropic_decay**	Soma-tropic force decays as a function of distance to this power
**inertial_force**	Maximum strength of forward growth bias due to stiffness: 0 indicates no bias, positive (unbounded) values introduce a bias
**branch_probability**	Global probability of bifurcation over one unit length of front. (Probability of branching at a front is equal to the probability of branching at least once over total front length; see section 2.3)
**front_extension**	Parameter for varying the length of a front's segments: 0 indicates “fixed” and otherwise “surface area dependent.” With positive surface area dependence, front segments becomes longer as their radius decreases; with negative, shorter
**TERMINATION CONDITION PARAMETERS**	
**bounding_shape**	Parameterized surfaces at which growing branches terminate
**intersection_proximity**	Minimum distance between an active front and any other segment
**max_fiber_length**	Maximum total fiber length in the neuron, at which all branches terminate
**max_bifurcations**	Maximum number of bifurcations in the neuron, at which all branches terminate
**min_radius**	Minimum segment radius, at which a branch terminates
**AUXILIARY PARAMETERS**	
**nbr_stems**	Number of soma branches
**start_radius**	Starting radius of each dendrite
**taper_rate**	Decay constant of segment radius over sequential fronts
**ralls_ratio**	Ratio of the sum of initial branch segment radii to their terminal parent branch segment radius
**angle_limit**	Lower an upper limit on the bifurcation and stem angles. Limits on bifurcation angle is rarely used but insures biological plausibility. Minimum of the uniform distribution of angles between inertial force vectors resulting from a bifurcation
**flatness**	Scaling factor for the *z*-dimension of 3-D Gaussian distributions from which growth directions are sampled: 0 generates a 2-D neuron, 1 generates a neuron with unconstrained 3-D morphology. (For flatness <0.5, bifurcation angles are sampled in the *xy* plane.)

### 2.2. Algorithm steps

After parsing the parameter text file, the C++ program that implements our algorithm first instantiates the soma with the specified surface area. The required neurite stems (soma branches) are created by iteratively sampling a random direction from the soma, calculating the angle to all other stems, and adaptively decreasing the minimum angle until an appropriate set of angles, and number of stems, is achieved. The algorithm then proceeds by repeatedly considering non-terminated branches (“fronts”) and extending each by either continuing or branching. Newly created branches are pushed onto the list of non-terminated branches (fronts). When continuing, the branch grows in a direction determined by adding a vector representing each directional bias to a sample from the 3-D Gaussian distribution, thus modeling the sum of the three homotypic forces.

When branching, two daughter branches are subjected to these biases, calculated separately for each. The algorithm revisits all non-terminated branches repeatedly until a neuron termination condition is reached. Termination conditions for branches include, in order of importance: (1) minimum segment diameter reached, (2) close proximity to another branch (measured surface to surface), and (3) collision with a fixed neuron boundary. Termination conditions for all neuron growth included, in order of importance: (1) all neuron branches terminated individually, (2) maximum total fiber length reached, and (3) maximum number of bifurcations reached. Finally, the morphology data is ordered and outputted according to the “swc” format (Cannon et al., 1998).

### 2.3. Implementation details

#### 2.3.1. The random process and force modeling

The influence of each of the three homotypic forces is modeled as an offset to the mean of a 3-D Gaussian distribution from which a direction of growth is sampled (Figure 1). For each dimension, the algorithm draws a sample from a Gaussian distribution multiplied by a constant standard deviation parameter for the neuron and summed with a variable offset for that dimension determined by the sum of local growth biases, where each unbiased sample, *s*, is calculated using Marsaglia's polar method (Marsaglia and Bray, 1964) as follows:

(1) $$s=\sigma v_{1}\sqrt{\frac{-2\ln(v_{1}^{2}+v_{2}^{2})}{v_{1}^{2}+v_{2}^{2}}},$$

where σ is the standard deviation and {*v*_1_, *v*_2_} ∈ *U*(−1,1)|0 < *v*^2^_1_ + *v*^2^_2_ < 1, where *U*(*a*, *b*) is the uniform distribution over the interval [*a*, *b*].

This process introduces stochasticity into our generative model of neuron morphology (Figure 2A). For certain morphologies (e.g., alpha motor neurons vs. Purkinje cells), the randomness of growth direction was increased by increasing the standard deviation of the neuron's 3-D Gaussian distribution. Furthermore, because of this stochasticity, a single parameter set could be used to generate a very large population of unique morphologies by varying each instance's random seed. (The resulting neurons have similar morphological traits and measures but are not identical.)

For each new segment added by continuing or branching at a front, a Gaussian distribution is centered on the front origin (see Equation 1). Next, a vector for the inertial force is calculated as a continuation of the previous segment, or as the direction of the randomly generated bifurcation angle. The magnitude of this vector is scaled by a user-specified bias-specific parameter, and the mean of the Gaussian displaced accordingly. A vector for the soma-tropic force is calculated similarly, representing the direction from the soma to the front origin, and additionally scaled by a user-specified function of this distance. Finally, a vector sum of the directions from all existing neuron segments to the front origin, with each direction vector scaled by a second user-specified function of distance, is calculated. With this vector sum, the mean of the Gaussian is displaced a third time. The center of the Gaussian may then be far from its initial mean.

Since only the direction is sampled from this Gaussian, the greater the displacement of the mean, the more likely the actual direction vector will be in the direction of the sum of the three forces, though this is never guaranteed since the Gaussian is of infinite extent. Finally, a segment is added in the sampled direction, and its length assigned according to the user-specified rate of growth. By adjusting these three forces, we show that the stochastic process may be biased to generate a variety of neuron morphologies (Figure 2B).

#### 2.3.2. Rate of growth and effects on morphology

Both continuing and branching at the end of a branch are conditioned by the rate of growth, which may be specified as a constant rate (in units of segment length per front, resulting in a pure Galton–Watson process), a rate which depends on the variable (tapered) radius (in units of segment surface area per front), or some intermediate rate. For variable growth rates, the Galton–Watson process is modified, such that branching occurs after a segment of growth according to the branching probability, conditioned by the segment's length. In this way, a *single* bifurcation point occurs at the end of each segment if the probability of *at least one* bifurcation point over that length exceeds a randomly generated number on the unit interval. Experimentally, it has been shown that in particular neuron types, the branching probability is non-uniform across the dendritic tree (Burke et al., 1992; Nowakowski et al., 1992). We found that certain non-uniform branching probabilities can be better approximated by our modified Galton–Watson process when we specify a non-uniform growth rate. In addition, because segments are straight, we found that certain neurons whose tortuosity decreases or increases with distance from the soma can also be approximated by specifying a non-uniform growth rate under constant influence from our three forces applied to segments of increasing length.

#### 2.3.3. 3-D vs. 2-D growth

Certain neuron types, such as retinal ganglion cells, starburst amacrine cells, and Purkinje cells, exhibit morphologies which are flat, covering a surface rather than filling a space. Our sampling procedure was initially developed for 3-D growth, but because growth depends entirely on sampling a 3-D Gaussian distribution, we were able to modify the procedure in a straightforward way in order to restrict growth to two dimensions by modifying the Gaussian distribution. More specifically, the sampling procedure was adjusted to include a user-specified scaling parameter applied to the standard deviation of the distribution's *z*-dimension. In this way, the direction of growth could be constrained in this dimension, or eliminated altogether (with a scaling factor of zero). In addition, we modified the procedure for determining the direction of inertial force vectors after branching, such that for scaling factors ≤0.5, bifurcation angles were restricted to the *xy* plane.

#### 2.3.4. Morphometric analysis

We analyzed the resulting morphologies' morphometric properties. In keeping with the aims of this work, we were most concerned with type-specific traits, such as the dimension of a morphology, the total length, the average path length to the terminal tips, and, space-filling proxies such as the fractal dimension and the contraction of branches. The fractal dimension is computed on a branch-by-branch basis by the Hausdorff fractal dimension (as described in Marks and Burke, 2007a): (〈*E*〉)^*D*^ = *t*, with 〈*E*〉 being the average increase in Euclidean distance per segment length and *D* the actual fractal dimension. A straight line always has the same Euclidean excursion from the soma per unit of path length and will result in *D* = 1, while a completely random walk will cover the surface (in 2-D) and have *D* = 2. Contraction is a similar metric indicating the ratio between the Euclidean distance and path length to a given terminal tip. The total number of branch points and terminal tips are also analyzed. For direct visual comparison of the topology we use dendrograms, and measured the asymmetry, *A*, of the tree of degree *n*, by summing over all previous branch points, *i*, each of which creates two subtrees with terminal segment counts *r* and *s*, according to van Pelt et al. (2001),

(2) $$A=\frac{1}{n-1}\sum_{i=1}^{n-1}A_{p}(r_{i},s_{i})$$

where *A*_*P*_ (*r*, *s*) ≡ |*r* − *s*|/(*r* + *s* − 2) for {*r*, *s*} ≠ {1,1}, and 0 otherwise. Finally, a Sholl-like analysis was performed to investigate the distribution (conditioned on branch order or path length) of selected morphometric properties (Sholl, 1953).

#### 2.3.5. Parallel implementation and availability

The compiled algorithm is very fast, with each neuron morphology presented here generated in <2 s using a 2.33 GHz microprocessor. For neural tissue simulation, in which thousands to millions of neurons may be generated in a single defined tissue volume (Kozloski, 2011), we anticipated that serial neuron morphogenesis will be too costly, and have therefore mapped our algorithm onto the parallel architecture of IBM's Blue Gene/P, such that each core of this massively parallel machine independently generates a unique set of neuron morphologies. By varying the random seed used to initialize this process on each machine core, we can produce a large space-filling tissue represented by a large number of swc files written in parallel to the machine's file system. These can then be read into a neural tissue simulator (Kozloski and Wagner, 2011) for subsequent physiological modeling. The final software is experimental. IBM would like to create an active user community for its neural tissue simulation tools. Readers are therefore encouraged to contact the corresponding author if interested in using the tool or in the source code.

## 3. Results

### 3.1. Morphological traits caused by homotypic forces

The influence of self-referential forces are manifested at both bifurcation points and continuing dendritic sections. As a reference, we generated a morphology in which no homotypic forces were present (Figure 2A). The resulting morphology resembles no neuron and instead represents a diffusion process (Brownian motion) modified by particle division and elimination according to our rules for branching and termination. We then added the three different self-referential forces at various levels of strength. The resulting morphologies are shown in Figure 2B. We observe that the introduction of homotypic forces unclutters the random morphology and brings structure to the shape. Moreover, the morphologies subjected to the homotypic forces resemble dendritic morphologies.

Next, we systematically investigated the morphological traits associated with a particular force. For the sake of clarity, we present neurons generated solely with 2-D growth, since the resulting dendritic trees are easier to inspect visually. Figure 3 summarizes these results. The strength parameter of each applied force is varied from 1 to 50 (arbitrary units; Table 1). For each force combination we generated 25 separate morphologies from different seeds to gather population statistics (although only one is depicted; Figure 3).

At the lowest level studied for each bias, we applied all three at strength 1 to impose structure (see Figure 2A). Therefore, morphologies in the first column of Figure 3 are similar as they all use the same force parameters (but different seeds). As a population these “baseline” structures have a total length of 2970 μm ± 800 and 230 ± 60 bifurcation points. The fractal dimension based on the Hausdorff metric is not always defined (Cannon et al., 1999), but the closely related contraction ratio was measured at 0.79 ± 0.05.

Next, we investigated the effect of increasing one of the three self-referential forces. First, in setting a higher inertial force parameter of 5 (top row in Figure 3), the total length initially increases to 4352 μ m ± 800 and 327 ± 63 branch points. At the highest parameter setting of 50, the size decreased again to slightly above the base line measurement (i.e., measurement when all force parameters set to 1). Then, we investigated the influence of the soma-tropic force and an entirely different morphology appears. With the highest soma-tropic force parameter used, the total length increases to 6388 μm ± 2299 and the morphology consists of 488 ± 176 branch points. The contraction increases to 1, which means that the dendrites follow a straight path directly away from the soma. Given the drastic increase in length when modeling the soma-tropic force rather than the inertial force, we can say that neurons subjected to this force form denser dendrites. Finally, when investigating the influence of the self-avoidance force, we see that the dendrites only become even denser. More specifically, with the highest tested self-avoidance parameter, the morphologies have a total length of 11,293 μm ± 1181 and 808 ± 144 branch points. The contraction at this level is 0.94 ± 0.2.

We thus observe that strong soma-tropism and strong self-avoidance results in larger structures that can potentially receive synaptic contacts from many more neurons. Moreover, with strong self-avoidance, the surface (space) is almost completely covered. This better coverage and larger structure directly results from self-avoidance among branches, which therefore grow and branch in parallel. The inertial bias alone also covers the surface (space) well, but less densely as the total length is considerably less when compared to the soma-tropic and self-avoidance examples. By means of this analysis, we conclude that sparse coverage of a space can be achieved by inertial forces, while dense coverage can be created by strong self-avoidance and strong soma-tropism.

### 3.2. Dendritic morphologies are shaped by homotypic forces

We established a relation between our modeled homotypic forces and the resulting morphologies of dendritic arbors. Do these traits also exist in real neuronal morphologies? We invert the question by generating dendritic morphologies exclusively based on homotypic growth biases. If the resulting morphologies resemble true morphologies, we can conclude that homotypic forces are sufficient to shape dendrites (though they may not do so exclusively). We generated three types of virtual neurons based on (and subsequently compared to) alpha motor neurons (cat), dentate gyrus granule cells (mouse), and Purkinje cells (mouse). We took experimentally reconstructed morphologies from the NeuroMorpho.org repository (Ascoli, 2006); the motor neurons were from Alvarez et al. (1998), the granule cells from Vuksic et al. (2008), and Purkinje cells from Martone et al. (2003). We chose motor neurons and granule cells are they are often the subject of algorithmic generation (Samsonovich and Ascoli, 2003; Marks and Burke, 2007b; Torben-Nielsen et al., 2008) and Purkinje cells because they have a peculiar and highly specific morphology. We adopted a strategy in which we tuned one parameter configuration to generate a virtual neuron that matches an exemplar reconstructed morphology. Then, due to the stochastic nature of the algorithm, we generated an arbitrary number of unique morphologies from this one configuration file using different random seeds.

**Figure 4:**
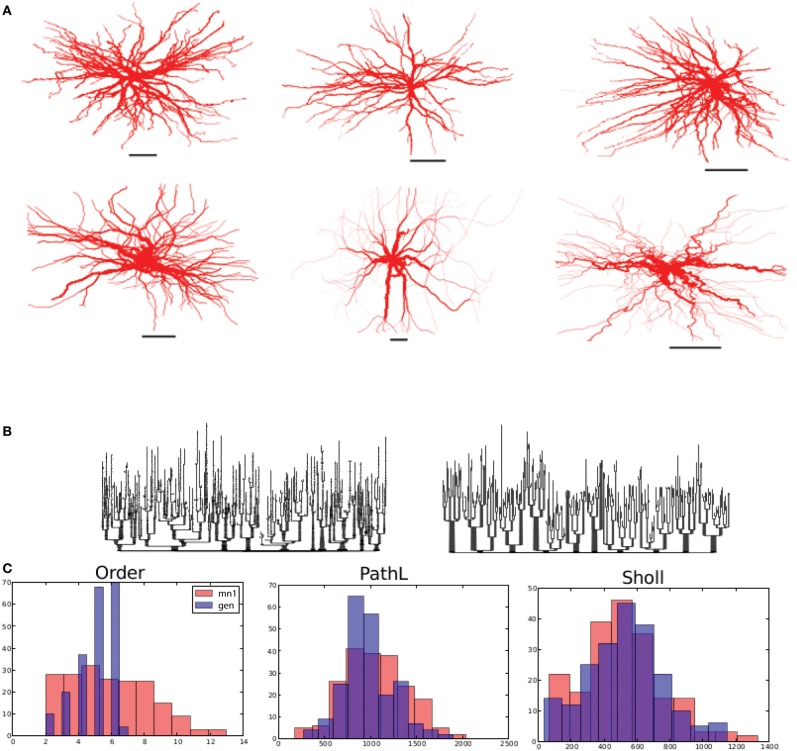
**Alpha motor neurons and their virtual counterparts. (A)** Reconstructed alpha motor neurons (from Alvarez et al., 1998) on the top row and generated virtual neurons on the bottom row. **(B)** Dendrogram of one reconstructed and one generated motor neuron on the left and right, respectively. **(C)** Comparison between reconstructed and generated motor neurons based on their measured distribution of the branching order, path lengths to terminal tips, and Sholl-intersections. Scale bars in **(A)** indicate 250 μm.

First, we investigated whether the motor neuron morphology could be dissected into influences of self-referential forces. To achieve a proper fit, we found a very strong soma-tropic growth bias that falls off rapidly with distance, together with a moderate self-avoidance bias (0.08–0.35) and a slightly stronger inertial bias (0.36–0.91) was sufficient. Figure 4A shows three reconstructed (top row) and three generated, virtual morphologies (bottom row). By visual inspection, the virtual morphologies appear plausible. To visualize the topology, we also constructed dendrograms of one reconstructed and one virtual motor neuron in Figure 4B. More quantitatively, we plotted the branch order distribution, the distribution of the path lengths (from each terminal tip to the soma), and the Sholl-intersections for one reconstructed and one virtual neuron (Figure 4C). While the distribution of the path lengths and the Sholl-intersections are similar, a clear difference can be observed between the branch order distribution of the virtual and reconstructed neurons. The mismatch in the order is a direct consequence of the simplified branching mechanism: we use a slightly modified Galton–Watson process that does not mimic the details of the true branch point distribution. Similarity between the Sholl-intersections and the distribution of path lengths indicates an overall similar pattern of outgrowth: the same space is occupied and the density of the dendritic trees are similar. The fractal dimension [on a branch to branch basis, as measured by the Hausdorff fractal dimension (Marks and Burke, 2007a)] is 1.09 ± 0.02 and 1.08 ± 0.02 for the reconstructed and generated motor neuron in Figure 4A (first column). The related contraction (i.e., Euclidean distance over path length) is 0.73 μm ± 0.08 and 0.77 μm ± 0.07 for reconstructed and generated morphologies, respectively. Together, fractal dimension and contraction are a good proxy for the space-filling properties of neurons. Therefore, we can observe that the generated morphologies fill the space to the same degree as the real motor neurons. Additionally, the bounding space is also set to match the space occupied by the exemplar reconstructed neurons and therefore the overall dimension of the generated morphologies matches well. The path length to the terminal tips is similar between experimentally reconstructed (792 μm ± 196) and generated morphologies (836 μm ± 214). Thus the geometry of the generated motor neurons matches the geometry of the exemplar reconstructed cells. Because of the good approximation of the overall geometry we can conclude that homotypic forces alone are sufficient to account for motor neuron geometry.

**Figure 5:**
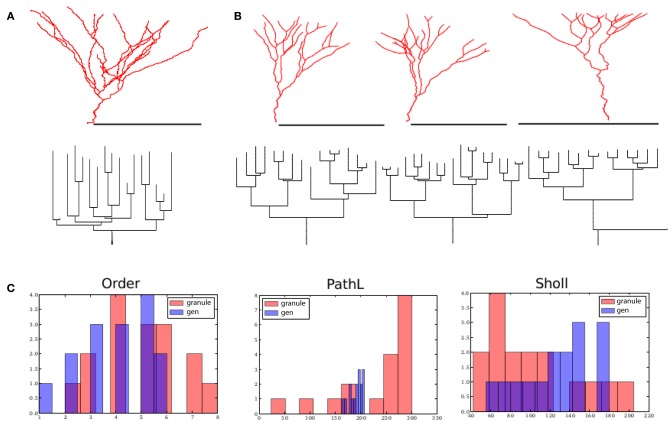
**Dentate gyrus granule cells and their virtual counterparts. (A)** Exemplar morphology from Vuksic et al. (2008) with its dendrogram. **(B)** Three morphologies generated from one parameter configuration each with its associated dendrogram. **(C)** Comparison between properties of one exemplar granule cell (from **A**) and one generated morphology (first from **B**). Scale bar in **(A)** and **(B)** indicate 100 μm.

Secondly, we set out to generate virtual granule cells. We tuned one parameter configuration that matched with GranuleCell-Nr3 from Vuksic et al. (2008). By generating morphologies from the same parameter set but with a different random seed, we could generate a set of unique morphologies matching well with the population of 20 exemplar cells. In comparison to alpha motor neurons, granule cells required a strong soma-tropic growth bias with a more gradual fall-off, a considerably stronger self-avoidance bias (4.5), and a comparable inertial bias (0.45). Strong self avoidance has also been demonstrated experimentally among Purkinje cell branches (Fujishima et al., 2012). In addition, the flatness parameter was reduced from 1.0 (fully 3-D) to 0.03 (strongly restricted to 2-D), since we observed that granule cells morphologies were partially flattened. A summary of the results is illustrated in Figure 5. The visual resemblance between the exemplar morphology and the generated morphologies is high. The number of terminal tips (14 ± 4) and bifurcation points (13 ± 4) is similar to the example cell (terminal tips 13 ± 3 and bifurcation points 12 ± 3). The total length of the generated granule cells, 1024 μm ± 373 (vs. 2068 μm ± 241), is slightly less for the generated morphology, while path length (205 μm ± 32) (vs. 225 μm ± 22) is comparable. The fractal dimension is much lower than for the motor neurons and amounts to 1 ± 0.02 for the generated cells and 1.01 ± 0.01 for the exemplar cells. From Figure 5 we can see that the distribution of the branching order and the Sholl-intersections are similar between the exemplar and generated cells. The path length, on average, is also similar but shows far less variance in the generated cells compared to the exemplar. However, based on the overall similarities, the shared dimensions, and the similarity in the space-filling property, we can conclude that granule cell morphology is sufficiently explained in terms of homotypic forces.

**Figure 6:**
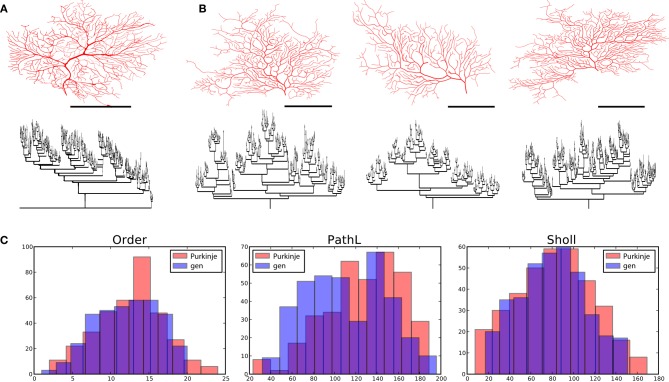
**Cerebellar Purkinje cells and their virtual counterparts. (A)** Exemplar morphology from Martone et al. (2003) with its dendrogram. **(B)** Three morphologies generated from one parameter configuration each with its associated dendrogram. **(C)** Comparison between properties of one exemplar Purkinje cell (from **A**) and one generated morphology (first from **B**). Scale bars in **(A)** and **(B)** indicate 100 μm.

Lastly, we generated a set of Purkinje cells based on one exemplar cell, namely e4cb2a2 from Martone et al. (2003) (Figure 6). Purkinje cell morphology is characterized by a large trunk from which smaller branches sprout. Also, the dendritic arbor densely covers the plane it occupies, thus requiring that smaller branches often grow toward the soma. We adjusted the parameter settings to match a generated morphology to that of e4cb2a2, then generated a set of different cells from these parameters using different random seeds. Specifically, we included a parameter that rendered branch diameters following a bifurcation unequal (while maintaining their specified Rall's ratio), and the algorithm maintained this asymmetry for much of the arbor. To achieve appropriate branch orders and good coverage, we also set the front extension parameter to a negative value, causing segment lengths to vary inversely with radius and thus branching frequency and tortuosity to slightly increase as the arbor expanded. In comparison to alpha motor neurons and granule cells, Purkinje cells required a 10–100 times greater branching probability, a moderate soma-tropic growth bias with a gradual fall-off, and both the strongest self-avoidance bias (5.4) and weakest inertial bias (0.32) among the three types. In addition, the flatness parameter was 0.0 (fully 2-D), since we observed that Purkinje cell morphologies were either fully within a plane, or occupied two distinct planes (a curious phenomenon our algorithm could not accommodate).

A quick visual inspection shows great correspondence between the exemplar morphology (Figure 6A) and the generated neurons (Figure 6B). The dendrograms are complex and difficult to compare, but the primary feature of a main trunk and a higher number of bifurcations combined with a high centrifugal order are observed. We then compared the complete set of four exemplar neurons available from the same lab through NeuroMorpho.org. The overall topology of the generated neurons compare well to the generated ones [373 ± 84 vs. 354 ± 11 terminal tips and partition asymmetry, Equation (2), of 0.44 ± 0.02 vs. 0.53 ± 0.01]. The geometry is also comparable in total length (generated 377 ± 918 vs. exemplar 5227 ± 544) and in path length (generated 108 ± 10 vs. 126 ± 14), albeit, the generated neurons being slightly smaller. This difference in size is in part due to the fact that the selected exemplar cell was the smallest of the set with a total length of 4461 μm. We find that the spatial embedding of the generated neurons as measured by the fractal dimension (generated 1.14 ± 0.05 vs. 1.09 ± 0.03) and the contraction (generated 0.74 ± 0.04 vs. 0.68 ± 0.02) also matches well with the exemplar cells. Curiously, the fractal dimension of the exemplar cells is lower than the generated cells indicating straighter branches in the exemplar cells while the contraction indicates exactly the opposite (stronger contraction for the exemplar cells). A summary of these data is also given in Figure 6C, where the match can also be visually evaluated.

## 4. Discussion

In this work we investigated the influence of homotypic forces on the morphology of dendrites. We followed a generative synthetic approach, in which artificial dendritic morphologies were solely determined by homotypic influences. By approximating three homotypic forces (inertial, soma-tropic, and self-avoidance) in a phenomenological growth model we could generate morphologies that have strong resemblance with real dendritic morphologies. We conclude that these homotypic forces are sufficient to explain dendritic morphology, though they may not do so exclusively. Consequently, we also argue that an estimate of these three homotypic forces should be used in future descriptions of dendritic morphology.

Despite a considerable amount of experimental evidence for external cues influencing dendritic morphology, there is little theoretical work on this topic. Moreover, in algorithmic approaches to generate whole morphologies, external influences are generally not considered. Recent noteworthy exceptions come from Samsonovich and Ascoli (2005), Luczak (2006), and Marks and Burke (2007a,b) to which we compare our work. Samsonovich and Ascoli set to dissect the main direction of growth in hippocampal pyramidal cell dendrites. They found that the main component was a soma-oriented tropism, and not a general gradient shared with other cells. Marks and Burke came to a similar conclusion and reported that, in motor neurons, the direction of the dendrites is both determined by the direction of the parent (i.e., what we call “inertial force”) and a soma-repulsive cue. Moreover, Marks and Burke also hinted at the influence of self-avoidance during the development of dendrites. An important distinction between first, the algorithms of Samsonvich and Ascoli and second, that of Marks and Burke, is that each starts with a dendrogram and only then adds a geometry. We generate both a topology (i.e., a dendrogram) and the spatial embedding (i.e., a geometry) at once, such that certain branching events resulted in one branch failing due to termination, while the other continues. Luczak used an algorithm based on diffusion limited aggregation that implicitly mimics precisely certain external cues (external gradients and self-avoidance). The results of Luczak indicate that a bounding box and competition over resources can account for dendritic-specific features that resemble self-referential and environmental interactions.

What is the relevance of the presented work? We can assess the relevance of the two pillars of our work, the branching rule based on a Galton–Watson process and homotypic forces defining the geometry. First, a Galton–Watson process is the simplest process to generate binary trees and relies on a constant branching probability independent of any other parameters in the algorithm. The simplicity of this process gives rise to discrepancies between true morphologies and the morphologies generated by our algorithm, namely the maximum branch order and distribution of bifurcations. With a pure Galton–Watson process, we would employ a constant branching probability (and hence a constant segment length independent of other variables and uniform across the whole tree). Several studies have indicated that the branching probability is not uniform, and can be dependent on the distance from a previous branch point (Nowakowski et al., 1992) or from the soma (Burke et al., 1992). Departing from our original intent, our algorithm may be modified in the future to generate highly realistic morphologies by substituting the Galton–Watson process with a more data-driven branching rule, or even a branching growth model as developed in van Pelt et al. (2001). The modified Galton–Watson process we used here did provide some ability to control a non-uniform branching probability, which we exploited to approximate more closely the properties of our real target neurons, such as their distributions of path lengths.

**Figure 7:**
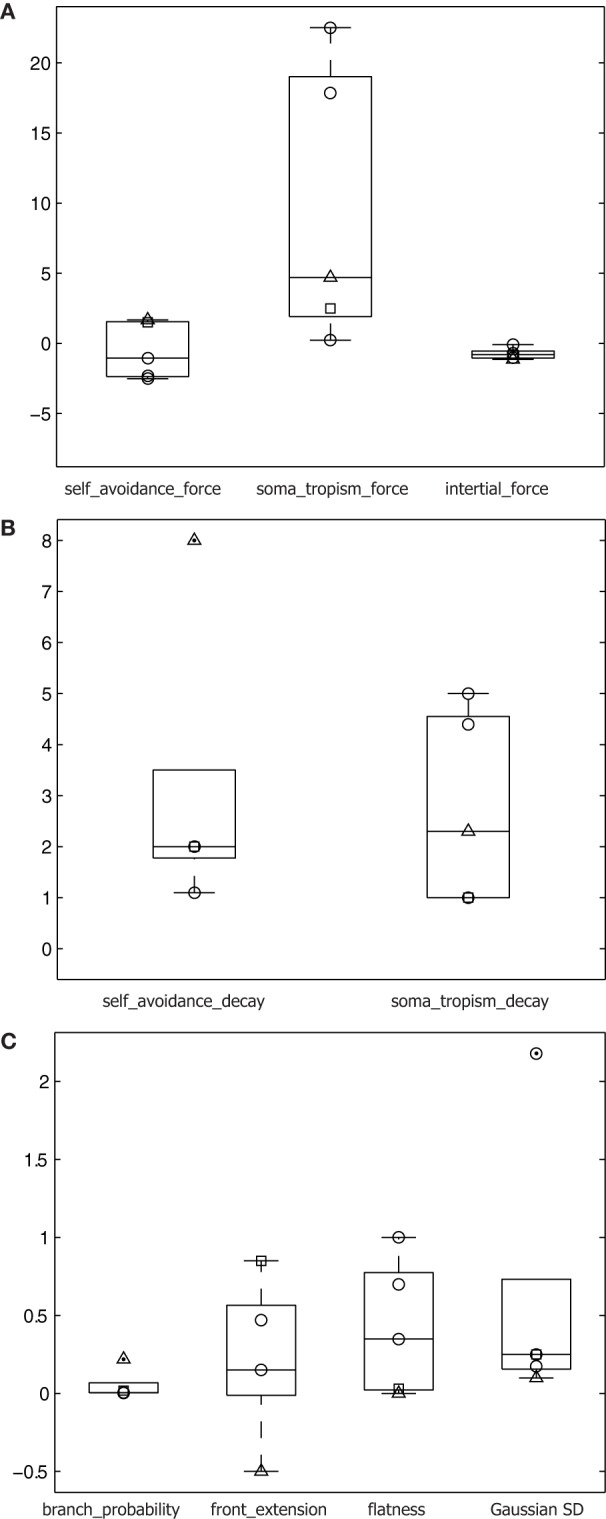
**Parameters differing across cell types. (A)** Force parameters plotted on logarithmic scale *y*-axis for the three cell types: alpha motor neurons (○), granule cells (□), and Purkinje cells (△). All parameters plotted and superimposed on box plots, with median indicated by a horizontal line, the box's edges indicating the 25th and 75th percentiles, the whiskers extending to all points not considered outliers, and outliers (>1.5 × the box height beyond the box edge) marked by a dot. **(B)** Spatial gradient parameters for the three cell types plotted on a linear scale *y*-axis. **(C)** Additional relevant parameters for the three cell types plotted on a linear scale *y*-axis.

Second, homotypic forces were modeled to guide the geometrical extent of the artificial dendrite morphologies, and varied greatly in comparison to other parameters across the different cell types we generated (Figure 7). Despite being a phenomenological model, the homotypic forces are biologically inspired. Stiffness resulting from an inertial force is documented (Condron and Zinn, 1997) and biases forward motion. Soma-tropic force has been exclusively described in a theoretical setting based on a transient gradient of pH induced by a cell's spiking activity (Samsonovich and Ascoli, 2005). Self-avoidance is also well documented based on observations of homotypic repulsion after actual contact (Jan and Jan, 2010; Fujishima et al., 2012) as well as from a distance (Lefebvre et al., 2012). In our model, repulsion follows a gradient such that actual contact between branches is not required. In addition, Shimono et al. (2010) and Sugimura et al. (2007) created detailed mechanistic models of growth at the molecular level, which also confirmed that self-repulsive effects are important in generating dendritic morphology. In contrast, the present study showed that simple models of self-referential forces can create realistic and diverse neuronal morphologies and did not attempt to capture the proximal physiological or molecular causes of these growth processes in a generative algorithm. Thus, the phenomenological incorporation of inertial, soma-tropic, and self-avoidance forces into our morphogenetic algorithm are grounded in the literature.

Of course there are also extrinsic actors (as opposed to self-referential or intrinsic forces) involved in shaping dendrites (Scott and Luo, 2001; Jan and Jan, 2010). These external influences can have a wide variety of sources. Neurons develop in a substrate populated by both other neurons and other structures such as blood vessels, capillaries and glia cells. Especially for optimal space coverage and tiling, interactions between cells seem required, and avoidance of other structures is evident. Moreover, neurons grow in a bounded space where the white matter, the pia, and even laminar structures can be seen as external influences on neurons. At a phenomenological level, these extrinsic actors can be described as forces in a (limited) spatial domain (as in Feng et al., 2005). Because we model the forces acting on the direction of growth as a sampling bias, we can easily extent the model to include other external forces in a similar fashion.

Our work demonstrates that self-referential forces are sufficient to explain the main morphological traits of adult dendrites. Experimental techniques, including genetic manipulation (Lefebvre et al., 2012), provide a means to access mechanistic causes of self-referential biases. As these techniques evolve, they may provide a means to test the predictions represented by our choice of parameters for those generated morphologies presented here, or for others derived from our algorithm. Our focus was not on generating the most realistic virtual neurons possible from a set of experimentally reconstructed morphologies. For example, as already discussed, our modified Galton–Watson branching process gives rise to a less realistic, nearly uniform branching frequency throughout the dendritic tree. In a similar vein, other specific features of dendritic morphology (e.g., segment length and branch angles) are not necessarily uniform across the tree (Burke et al., 1992; Nowakowski et al., 1992; Torben-Nielsen et al., 2008; Langhammer et al., 2010), and instead vary as a function of some topological measures of the tree (such as path length, branch order, etc.). Therefore, a potential extensions to this work, aimed at the generation of realistic morphologies, could include other parameters (such as those describing homotypic forces and their influence) that vary over the dendrite in this way.

Recently it was also reported that external guidance cues are not required for synaptic patterning and contact formation (Hill et al., 2012). While the finding is surprising, it does not speak to the need for intrinsic or extrinsic cues during development of the morphology itself. Moreover, it is reasonable to think that evolution caused a particular trait in a population, giving rise to a statistically relevant number of appositions between dendrites and axons, to become the basis for regular cell-to-cell wiring and communication in neural tissues within a species. This result also implies that neurons can, in principle, be generated independently from each other, as long as they share population statistics with a measured population of real neurons. In our study we managed to dissect morphological traits of dendrites stemming from the interaction and influence of three self-referential forces. In combination with adequate branching rules, our algorithm provides a parsimonious description to generate vast numbers of virtual neurons. Moreover, we speculate that morphologies from many distinct classes exhibit morphological traits that result from self-referential interactions biasing an otherwise random process. Tuning the small set of parameters underlying our algorithm is more straightforward than tuning an exhaustive set of detailed, local parameters (such as the distribution of branching angles, which in turn might be conditioned on various other dendritic parameters such a diameter and distance from the soma). Thus, our insights into the growth of single neurons could be used to generate large populations of neurons and synapses for large-scale computer simulations of neural tissue.

### Conflict of interest statement

The authors declare that the research was conducted in the absence of any commercial or financial relationships that could be construed as a potential conflict of interest.
